# Optimized Gamma Synchronization Enhances Functional Binding of Fronto-Parietal Cortices in Mathematically Gifted Adolescents during Deductive Reasoning

**DOI:** 10.3389/fnhum.2014.00430

**Published:** 2014-06-11

**Authors:** Li Zhang, John Q. Gan, Haixian Wang

**Affiliations:** ^1^Key Laboratory of Child Development and Learning Science of Ministry of Education, Research Center for Learning Science, Southeast University, Nanjing, China; ^2^School of Computer Science and Electronic Engineering, University of Essex, Colchester, UK

**Keywords:** mathematically gifted adolescents, fronto-parietal functional binding, EEG cortical network, gamma phase-locking duration, power-law model

## Abstract

As enhanced fronto-parietal network has been suggested to support reasoning ability of math-gifted adolescents, the main goal of this EEG source analysis is to investigate the temporal binding of the gamma-band (30–60 Hz) synchronization between frontal and parietal cortices in adolescents with exceptional mathematical ability, including the functional connectivity of gamma neurocognitive network, the temporal dynamics of fronto-parietal network (phase-locking durations and network lability in time domain), and the self-organized criticality of synchronizing oscillation. Compared with the average-ability subjects, the math-gifted adolescents show a highly integrated fronto-parietal network due to distant gamma phase-locking oscillations, which is indicated by lower modularity of the global network topology, more “connector bridges” between the frontal and parietal cortices and less “connector hubs” in the sensorimotor cortex. The time domain analysis finds that, while maintaining more stable phase dynamics of the fronto-parietal coupling, the math-gifted adolescents are characterized by more extensive fronto-parietal connection reconfiguration. The results from sample fitting in the power-law model further find that the phase-locking durations in the math-gifted brain abides by a wider interval of the power-law distribution. This phase-lock distribution mechanism could represent a relatively optimized pattern for the functional binding of frontal–parietal network, which underlies stable fronto-parietal connectivity and increases flexibility of timely network reconfiguration.

## Introduction

In the fields of education and psychology, exceptional logical reasoning and visual-spatial imagery abilities are regarded as the main characteristics of mathematically gifted adolescents. Numerous neuroscience studies have reached an agreement that the gifted mathematical thinking abilities are supported by a cooperative fronto-parietal network (O’Boyle et al., 2005; Lee et al., 2006; Wartenburger et al., 2009; Prescott et al., 2010; Desco et al., 2011; Hoppe et al., 2012), including the widespread activation of fronto-parietal cortices, the heightened intrahemispheric frontal–parietal connectivity, and the enhanced interhemispheric frontal connectivity between the dorsolateral prefrontal and premotor cortices (Prescott et al., 2010). Some empirical studies have further suggested that the functional facilitation of the fronto-parietal network is driven by the extensively activated posterior parietal cortices (Lee et al., 2006; Desco et al., 2011). Besides, math-gifted adolescents were found having a larger number of fronto-parietal connections within the right hemisphere as compared with the left hemisphere (Prescott et al., 2010). Based on the highly developed right hemisphere and well-developed interhemispheric interaction, math-gifted adolescents can activate a “bilateral” fronto-parietal network during the cognitive processing related to mathematical thinking (Alexander et al., 1996; Sternberg, 2003; O’Boyle et al., 2005; O’Boyle, 2008; Desco et al., 2011). Therefore, the heightened “interplay” of anterior/posterior accompanied with the enhanced interhemispheric frontal connectivity, the extensive parietal activation and the bilateral fronto-parietal network have been suggested as the important neural mechanisms of the math-gifted brain (Singh and O’Boyle, 2004; O’Boyle et al., 2005; Lee et al., 2006; Prescott et al., 2010; Desco et al., 2011).

The parieto-frontal integration theory (P-FIT) on individual differences in reasoning competence emphasizes the crucial process of information communication between association cortices within the parietal and frontal brain regions (Jung and Haier, 2007). Neural oscillations and synchronization represent important mechanisms for interneuronal communication and binding of information among distributed brain regions. Specifically, gamma oscillations (30–60 Hz) are considered as the important building blocks of the electrical activity of the brain and possibly represent a universal code of information communication in the central nervous system (Basar et al., 1999, 2001). Gamma-band modulation in spectral power shows spatial correspondence with the fMRI blood oxygenation level dependent (BOLD) variation in the activated regions of the brain (Niessing et al., 2005; Lachaux et al., 2007). Gamma oscillation is also highly involved in sensation, perception, and cognition, and is correlated with high-order cognition, working memory load, and decision-making, etc. (Karakas et al., 2001; Howard et al., 2003; Fitzgibbon et al., 2004; Herrmann et al., 2010). As low-frequency oscillations coordinate long-range functional connectivity, gamma synchronization oscillation is more spatially restricted and reflects high-density local information processing (Brovelli et al., 2005; Bassett et al., 2006), which has been proposed as a crucial mechanism for the short-lasting functional binding between discrete brain regions (Koenig et al., 2005). Furthermore, the gamma binding-by-synchrony activity among neuronal populations constitutes a transient, large-scale, and task-specific functional neurocognitive network (Basar-Eroglu et al., 1996; Doesburg et al., 2008; Uhlhaas et al., 2011).

On the other hand, the network with dynamic binding not only depends on the transient coupling between neural assembles, but also requires the timely reconfiguration of connections to adapt to external stimuli and inner perturbation. As a representation of functional coupling strength between adjacent or distant brain areas, the synchronization between neuronal assembles is actually operated in a metastable dynamic system (Werner, 2007). For example, EEG phase synchronization (PS) is a mixture of episodic phase-locking durations interrupted by phase-shifts (desynchronization) in spontaneous EEG (Freeman and Rogers, 2002; Chialvo, 2004; Thatcher et al., 2009a). As continuous phase-locks enhance the functional coupling between neuronal populations and lead to the emergence of connections in neuronal networks, phase-shifts mark the beginning of a different set of connections and the occurrence of network reconfiguration (Thatcher, 2012). Moreover, these phase-locking durations have been discovered to conform the rule of power-law distribution, which has been widely accepted as a typical empirical signature of non-equilibrium systems in self-organized critical states (Kitzbichler et al., 2009). The gamma network in particular has been found having the highest global synchronizability in the fractal networks of the brain, suggesting that the gamma synchronizing network is dynamically located at a critical edge in transit to desynchronization. The highly critical state of the gamma network increases its adaptiveness to cater for changing environmental requirements through rapid reconfiguration of connections (Bassett et al., 2006).

Through EEG source analysis of the gamma cortical network, the present study aims to find the giftedness-related capacity of functional binding in the crucial fronto-parietal network of reasoning, by assessing the task-related functional connectivity and adaptive network reconfiguration. The study first compared the basic cortical network topologies constituted by gamma phase-locking oscillations in math-gifted and average-ability adolescents while they were performing a deductive reasoning task. Furthermore, at a neural-mechanistic level of analysis, the study investigated the temporal dynamics of the fronto-parietal network, including the phase-locking intervals/durations (PLI) and the lability of fronto-parietal network reorganization. Then, the parameter fitting of the PLIs in the power-law model was conducted to assess the criticality of phase-locking durations, which could construct an association between the functional connectivity and adaptive reconfiguration of fronto-parietal network. After that, the relationship among the enhanced fronto-parietal connectivity, the extensive reorganization of fronto-parietal connections, and the high criticality of PLIs in the math-gifted brain was analyzed and discussed.

## Materials and Methods

### Subjects

Two groups of subjects were enrolled in this study. The math-gifted group included 11 adolescents (eight males and three females) aged 15–18 years (mean ± SD: 16.3 ± 0.6), who were from the Science and Engineering Experimental Class at Southeast University (Nanjing, China). The class was composed of adolescents who had been recruited through a special college entrance examination aiming at gifted students under 15 years old with exceptional abilities in mathematics and natural sciences. Three criteria were employed to select math-gifted subjects from the class according to the definition of “school giftedness” (Renzulli, 1978; Heller, 1989): (1) nomination: they were recommended by their teacher according to their behavioral performance; (2) academic performance: they should have been awarded prizes in nationwide or provincial mathematical competitions; (3) intelligence score: their scores of Raven Advanced Progressive Matrices (RAPM) test were higher than 32 (mean ± SD: 33.6 ± 0.8). For the control group, 13 subjects were recruited from the Fourth High School of Nanjing, using the following criteria: (1) they were matched with the math-gifted group for age (mean ± SD: 15.9 ± 0.7) and gender (eight males and five females); (2) they had average-level performance in mathematical class tests; (3) their scores of RAPM test were <32 (mean ± SD: 23.5 ± 4.5).

The exclusion criteria adopted included left handedness, medical, neurological or psychiatric illness, and history of brain injury or surgery. To avoid the long-term training effect on the human brain activity, students who had received special training course of Mathematical Olympiad were excluded from this experiment. All the subjects were given informed consent and the study was approved by the Academic Committee of the Research Center for Learning Science, Southeast University, China. The subjects received financial compensation for their participation.

### Experimental paradigm

As the essential mathematical skill and the standard type of deductive reasoning, a categorical syllogism task of analytic type (verbal–logical way) was adopted in this study. Categorical syllogism is constituted by a major premise, a minor premise, and a conclusion. The actual reasoning process has been considered to emerge during the presentation of the minor premise and remain active until the validation of the conclusion (Fangmeier et al., 2006; Rodriguez-Moreno and Hirsch, 2009). Neuroimaging studies have identified that frontal, parietal, temporal, and occipital complexes are involved in deductive reasoning tasks (Goel et al., 2000; Goel and Dolan, 2001; Knauff et al., 2002; Goel, 2007). Particularly, the activations in the left inferior frontal gyrus, bilateral precentral gyrus of the left fronto-parietal system, and the left basal ganglia have been consistently reported to be specific to categorical syllogism (Prado et al., 2011).

**Figure 1:**
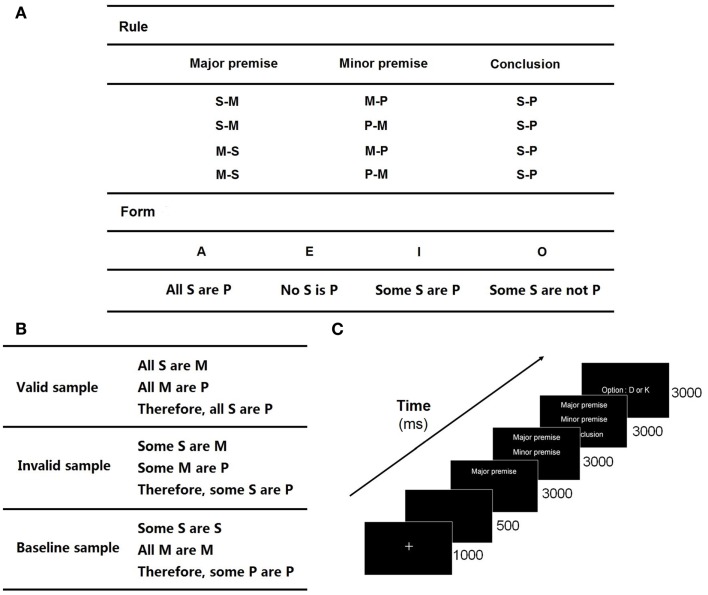
**Experiment protocol**: **(A)** rules and forms for cross-combination in logical syllogism. The valid forms utilized in this experiment are AAA, AII, EAE, EIO, AEE, EAE, EIO, AOO, AAI EAO, IAI, OAO, which are adapted from Evans et al. (1993). For example, a valid combination of EAE and the first rule is “No S is M; All M are P; Therefore, No S is P”; **(B)** some samples of valid, invalid, and baseline trials; **(C)** timeline of the stimuli.

The syllogistic sentences without specific content include three basic items: “S,” “M,” and “P.” “M” is the medium item and is presented in both the major premise and the minor premise. “S” and “M” constitute the major premise, and “M” and “P” the minor premise. From the two premises, the inferred relationship between “S” and “P” forms the conclusion (Figures 1A,B).

The experiment adopted a three-block paradigm, included a valid block (32 trials), an invalid block (32 trials), and a baseline block (40 trials). The combinations of syllogistic sentences following the true logical rules constituted a valid block, which employed the logic expressions proposed by Evans et al. (1993). An invalid block was constituted by the invalid combinations of syllogistic sentences, in which there was inconclusive relationship between two premises or incorrect conclusion under clear premises. A baseline block consisted of the trials including the same letter items in each sentence, in which there was no need for subjects to think of the relationship between the items. The letters used in the syllogistic sentences were randomly selected from the 26 letters of the English alphabet. Some samples are shown in Figure 1B.

The trials of the three blocks were presented in a random order, which was performed by the E-Prime 2.0 experimental procedure. The stimuli presentations of all the trials took about 30 min. The major premise, minor premise, and conclusion were presented sequentially along the timeline (Figure 1C). When the minor premise was shown, subjects were asked to draw a logical conclusion to judge whether the subsequent conclusion was valid or invalid (the ratio of the numbers of valid and invalid trials was 1:1). Subjects put their left index finger on “D” key and right index finger on “K” key at the beginning of a trial. They were asked to respond as accurately as possible by pressing “D” for “invalid” and “K” for “valid” within 3000 ms after the presentation of the conclusion. The time length of a reasoning process is 9000 ms.

Before the formal experiment, a practice session including five trials was conducted by each subject. After that, they decided whether to practice again or enter the following formal procedure. The sentences included Chinese characters and English letters, which were white on black background to avoid visual fatigue.

### EEG recording and preprocessing

**Figure 2:**
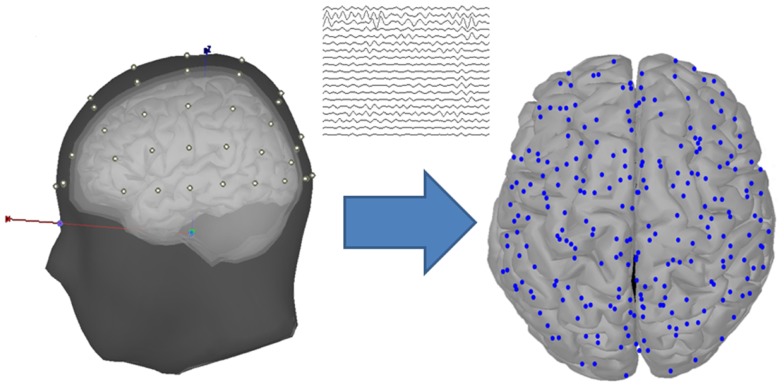
**Head model and cortical vertices**. The diagram located at the left of the arrow shows the head model with four layers (scalp, outer skull, inner skull, and cortex), and the scalp is placed with 60 EEG data channels. The diagram located at the right of the arrow shows the cortical vertices that are transformed from the EEG channel locations through a source estimation procedure.

The EEG data were recorded using the Neuroscan system at sampling rate 1000 Hz, with 60 scalp electrodes placed according to the international 10–20 system (Figure 2). Additionally, bilateral mastoids were used to place the reference electrodes. To monitor ocular movements and eye blinks, electro-oculographic (EOG) signals were simultaneously recorded by four surface electrodes, with one pair placed over the higher and lower left eyelids and the other pair placed 1 cm lateral to the outer corners of the left and right orbits.

By using the Scan 4.3 data preprocessing software, the continuous EEG signals with correct responses were band-pass filtered between 1 and 100 Hz. The epoch of each trial was extracted using a time window of 9500 ms (500 ms pre-stimulus and 9000 ms post-stimulus), and was baseline-corrected according to the pre-stimulus time interval. Ocular artifacts were removed according to the simultaneously recorded EOG signals. After the artifact rejection with the thresholds ranging from 50 to 75 μV, the blink and electrocardiogram noises were excluded. Finally, 18–22 trials were retained for each math-gifted subject and 15–25 trials were retained for each control subject. In addition, the independent component analysis (ICA) in the EEGLAB Toolbox was used to further clear the visible artifacts, such as the components of possible ocular and muscle movements. Since the emergence of the minor premise in the syllogistic sentence was viewed as the beginning of the actual reasoning process, the time interval 3000–9000 ms (presentation time of the minor premise and conclusion) of the artifact-free EEG signal was selected as the event-related time window. Because of the individual differences in response speed and completion time of each trial, the interval 4000–8000 ms was further extracted as the time window for data analysis.

### Gamma-band response and cortical source estimation

#### Gamma-band response

Task-induced response at the gamma frequency of the human brain activity was first assessed in each EEG channel by calculating event-related synchronization/desynchronization (ERS/ERD), which was expressed as the percentage of power increase/decrease relative to the baseline resting state:

(1) $$\text{ERS/ERD }\left(f\right)=\left[A\left(f\right)-R\left(f\right)∕R\left(f\right)\right]\times 100\%$$

where *f* indicates the gamma frequency band, *A*(*f*) is the power spectrum density (PSD) of an EEG signal in the task period and *R*(*f*) is the PSD in the pre-stimulus interval of the signal. Positive value is ERS and negative value represents ERD.

#### Cortical current estimation

There is a limitation that the EEG-based brain connectivity analysis was influenced by the volume conduction, which was caused by the variation of the electrical conductivity among the different head layers (Langer et al., 2012; Klados et al., 2013). To avoid this problem, the scalp-recorded EEG signals were transformed into the source space, which was performed by using the source estimation procedure of the Brainstorm Toolbox that is documented and freely available at http://neuroimage.usc.edu/brainstorm (Tadel et al., 2011). In the source estimation, the EEG signals were assumed to be mainly determined by a block of electric dipoles located at the surface of the cortex. Based on an averaged realistic head model that was constituted by four layers, i.e., scalp, outer skull, inner skull, and cortex, the symmetric Boundary Element Method (BEM) in the open-source software (http://www-sop.inria.fr/athena/software/OpenMEEG/) (Gramfort et al., 2010) was applied to the EEG electrode locations to obtain the volume conductor modeling of the subjects, i.e., the forward model matrix. Through an inverse kernel matrix produced by the standardized Low Resolution Brain Electromagnetic Tomography (sLORETA) and the forward model, the raw EEG signals were transformed into the current sources located at the cortical surface. By applying a downsampling procedure to the original sources, 248 cortical vertices were selected to serve as the nodes in the following graph theory analysis (Figure 2).

### Phase synchronization and undirected graph construction

To quantify the strength of connectivity, the cortical currents were followed by a phase-locking value (PLV) calculation between each pair of the nodes. PLV is a representative method of PS through obtaining a statistical quantification of the frequency-specific synchronization strength between two neuroelectric signals (Lachaux et al., 1999). The phase-locked neuronal groups can communicate effectively, because the communication windows between these neuronal populations for input and output are open at the same time (Fries, 2005). For two signals *x*(*t*) and *y*(*t*) with instantaneous phases *ϕ_x_*(*t*) and *ϕ_y_*(*t*), PS is the locking of the phases associated to each signal, i.e., |*ϕ_x_*(*t*) − *ϕ_y_*(*t*)| = const. Phase can be obtained through the Hilbert transform (HT), which is used to constitute an analytical signal as $H\left(t\right)=x\left(t\right)+i\tilde{x}\left(t\right)$. Here, $\tilde{x}\left(t\right)$ is the HT of *x*(*t*), defined as $\tilde{x}\left(t\right)=\frac{1}{\pi }\text{PV}\int_{-\infty }^{\infty }\frac{x\left(t^{\prime }\right)}{t-t^{\prime }}dt^{\prime }$, where PV denotes the Cauchy principal value. The phase of the signal *x*(*t*) is defined by $\phi_{x}\left(t\right)=\arctan\;\tilde{x}\left(t\right)∕x\left(t\right)$. The PLV bivariate metric for *ϕ_x_*(*t*) and *ϕ_y_*(*t*) is defined as

(2) $$\text{PLV}=\left|\frac{1}{M}\sum_{j=0}^{M-1}\exp\;\left(i\left(\phi_{x}\left(j\Delta t\right)-\phi_{y}\left(j\Delta t\right)\right)\right)\right|$$

where Δ*t* is the sampling interval and *M* is the number of sample points of each signal. The range of PLV is within [0,1], where 1 denotes perfect PS and 0 represents absence of synchronization (Sakkalis, 2011).

After calculating the PLV matrix of size 248 × 248 for all the cortical vertices, a fixed connection density was employed to acquire the adjacency matrix. The connection density of the network was set to *p* = 21n *n*/*n* according to the Erdos–Renyi model (Erdos and Renyi, 1961), where *n* is the number of the nodes. After that, the graph theory was used to quantify the topological properties of the adjacency matrix (Bullmore and Sporns, 2009; Rubinov and Sporns, 2010).

In the following definitions of the graph-theoretical measures based on an adjacency matrix [*a_i_*_,_*_j_*], *N* is the set of all the nodes in a functional brain network (*i*, *j*) represents the link between nodes *i* and *j* (*i*, *j* ∈ *N*). If there is a link (*i*, *j*) between nodes *i* and *j*, then *a_i_*_,_*_j_* = 1, which denotes a connection status; otherwise, *a_i_*_,_*_j_* = 0.

*Degree* of node *i* is the number of links connected to it:

(3) $$k_{i}=\sum_{j\in N}a_{ij}$$

*Modularity* of a network is defined by

(4) $$Q=\sum_{u\in M}\left[e_{uu}-{\left(\sum_{\nu \in M}e_{u\nu }\right)}^{2}\right]$$

where *M* is a set of non-overlapping modules that the network can be fully divided. *e_uv_* is mainly determined by the ratio of the number of the links connecting the nodes in module *u* with the nodes in module *v* to the total number of the links in the network.

*Characteristic path length* is defined by

(5) $$L=\frac{1}{n}\sum_{i\in N}L_{i}=\frac{1}{n}\sum_{i\in N}\frac{\sum_{j\in N,j\neq i}d_{ij}}{n-1}$$

where *L_i_* is the average distance between node *i* and other nodes, and *d_ij_* is the shortest path length between nodes *i* and *j*, which is given by $d_{ij}=\sum_{a_{mn}\in g_{i\to j}}a_{mn}$ (*g_i_*_→_*_j_* is the shortest geodesic path between *i* and *j*. For all disconnected pairs *i*, *j*, *d_ij_* = ∞).

*Node clustering coefficient* is quantified by a proportion of the number of existing connections between the nearest neighbors of a node *i* to the number of maximally possible connections:

(6) $$C_{i}=\frac{2t_{i}}{k_{i}\left(k_{i}-1\right)},\;\left(C_{i}=0\;\text{if}\;k_{i}<2\right),$$

where *t_i_* is the number of triangles around node *i*, i.e., $t_{i}=\frac{1}{2}\sum_{j,h\in N}a_{ij}a_{ih}a_{jh}$, *k_i_* is the degree of the node.

*Node betweenness centrality* is measured according to the proportion of the number of the shortest paths between all the node pairs passing through a specific node to the total number of shortest paths between all the node pairs, which can assess the communication role of the node within the functional network and is defined as follows:

(7) $$b_{i}=\frac{1}{\left(n-1\right)\left(n-2\right)}\sum_{\begin{array}{c} h,j\in N\\ h\neq j,h\neq i,j\neq i \end{array}}\frac{\rho_{hj}\left(i\right)}{\rho_{hj}}$$

where *ρ_hj_* is the number of the shortest paths between nodes *h* and *j*, and *ρ_hj_*(*i*) is the number of the shortest paths between nodes *h* and *j* that pass through node *i*. A node with high betweenness centrality is thus crucial to play the role of “connector hub” in the network.

*Edge betweenness centrality* is calculated based on how many of the shortest paths between all the node pairs in the network pass through a specific edge:

(8) $$B_{ij}=\frac{1}{\left(n-1\right)\left(n-2\right)}\sum_{\begin{array}{c} h,k\in N\\ i\neq j,h\neq k\\ h\neq i,h\neq j\\ k\neq i,k\neq j \end{array}}\frac{\rho_{hk}\left(ij\right)}{\rho_{hk}},\;\left(a_{ij}=1\right)$$

where *ρ_hk_* is the number of the shortest paths between nodes *h* and *k*, and *ρ_hk_*(*ij*) is the number of the shortest paths between nodes *h* and *k* passing through edge (*i*, *j*). An edge with high betweenness centrality represents a “connector bridge” between two parts of a network, the removal of which might affect the communication between many pairs of nodes through the shortest paths between them.

### Phase-locking duration and network lability during dynamic binding process

Since PLV is the temporal statistic of the intermittent phase-locking durations in a specified time interval, the PLIs between frontal and parietal cortical signals were extracted to further quantify the distribution characteristic of the continuous synchronizations. PLI is the period of time when two oscillators maintain the synchronization activity in their phase difference within a limited range, i.e., Δ*ϕ_xy_*(*t*) = |*ϕ_x_*(*t*) − *ϕ_y_*(*t*)| < const. In this paper, PLI is defined as the length of time during which two signals *x*(*t*) and *y*(*t*) are synchronized by satisfying the condition of $-\frac{\pi }{4}<\Delta \phi_{xy}\left(t\right)<\frac{\pi }{4}$ (Kitzbichler et al., 2009). If this condition does not hold true, the phase-locking oscillation is interrupted and enters into the phase-shifting interval.

On the other hand, to measure the coordinated change of functional coupling states of the synchronizing network during reasoning task, the fronto-parietal lability was calculated in the selected nodes ranging from frontal, sensorimotor to parietal cortices. The lability is quantified by the total number of phase-locking pairs of signals in a dynamic network that can change over time. The number of signal pairs that were phase-locked at any time points can be acquired according to the following preset condition of phase difference:

(9) $$N\left(t\right)=\sum_{x<y}b\;\left(\left|\Delta \phi_{xy}\left(t\right)<\frac{\pi }{4}\right|\right)$$

where $b\left(\left|\Delta \phi_{xy}\left(t\right)\right|<\frac{\pi }{4}\right)=\left\{\begin{array}{cc} 1, & \text{if}\;\left|\Delta \phi_{xy}\left(t\right)\right|<\frac{\pi }{4}\\ 0, & \text{otherwise} \end{array}\right.$

The lability of a synchronizing network is defined as

(10) $$\Delta^{2}\left(t,\;\Delta t\right)={\left|N\left(t+\Delta t\right)-N\left(t\right)\right|}^{2}$$

where the time interval Δ*t* was set to 10, 15, 20, and 25 ms respectively, as 10–30 ms had been proposed as the optimal temporal window for information transmission and storage in cortical circuits (Harris et al., 2003). It is clear that larger Δ^2^(*t*, Δ*t*) represents more extensive change in the fronto-parietal network and more flexible adjustment of the functional connections.

For all the trials, the scattergrams were constituted by the samples with the feature distribution of mean fronto-parietal PLI and mean lability of fronto-parietal network in 10, 15, 20, and 25 ms time intervals. Linear discriminant analysis (LDA) (Webb, 2003) with 10-fold cross-validation was employed to further discover the giftedness-related dynamic functional binding pattern.

### Criticality assessment of phase-locking durations

To construct an association between PLI and functional reorganization of network, critical dynamics of the fronto-parietal synchronization is assessed by fitting the PLIs in the “power-law” model. The PLI distributed in a critical interval indicates that a “metastable” synchronization is in effect, which implies the synchronizing state would access “neuronal avalanche” and adaptive reorganization by synaptic interaction in the face of endogenous perturbation and external event (Werner, 2007; Beggs, 2008; Kitzbichler et al., 2009; Thatcher et al., 2009a).

Playing the role of functional integration between posterior parietal and frontal cortices in reasoning (Jung and Haier, 2007), the inter-module connections between frontal and parietal cortical areas are crucial for straightforward coupling. Therefore, the phase-locking durations between 30 × 30 frontal–parietal node pairs were concatenated to constitute the inter-node PLI sample set.

In this study, the parameter fitting method proposed by Clauset et al. was applied to the PLIs set. The method has been proven valid on various datasets from the natural phenomenon with power-law distribution characteristic (Clauset et al., 2009). Let *x* represents a discrete set of PLI values, a discrete power-law distribution can be described by the following probability density

(11) $$p\left(x\right)=P_{\text{r}}\left(X=x\right)=Cx^{-\alpha }$$

where *X* represents the observed PLI value, *C* is a normalization constant, and *α* indicates the power-law exponent. It is clear that smaller *α* indicates a higher probability of long phase-locking duration. In practice, not all the PLI values obey the power-law, and only the values greater than a minimum value *x*_min_ can fit in the power-law distribution with less bias. While the data are drawn from a distribution that follows a power-law exactly for *x* ≥ *x*_min_, the scaling parameter *α* can be estimated correctly. In the special case of *x*_min_ = 1, the maximum likelihood estimator (MLE) used for appropriate estimation of *α* is given by the solution to the transcendental equation $\frac{\zeta \prime \left(\hat{\alpha }\right)}{\zeta \left(\hat{\alpha }\right)}=-\frac{1}{n}\sum_{i=1}^{n}\ln\;x_{i}$, where ζ is the Riemann zeta function. When *x*_min_ > 1, the zeta function is replaced by the generalized zeta $\frac{\zeta \prime \left(\hat{\alpha },\;x_{\min}\right)}{\zeta \left(\hat{\alpha },\;x_{\min}\right)}=-\frac{1}{n}\sum_{i=1}^{n}\ln\;x_{i}$. For each possible choice of *x*_min_, *α* was estimated by the MLE. The Kolmogorov–Smirnov (KS) goodness-of-fit statistic was calculated according to $D=\underset{x\geq x_{\min}}{\max}\;\left|S\left(x\right)-P\left(x\right)\right|$, where *S*(*x*) is the cumulative distribution function of the data for the observation with the value larger than *x*_min_, and *P*(*x*) is the cumulative distribution function of the best fitting of data to the power-law model in the region *x* ≥ *x*_min_. The optimal estimation of *x*_min_ is the one that gives the minimum value of *D*. Root-mean-square error (RMSE) expressed by $R_{\text{e}}=\sqrt{\left[\sum {d_{i}}^{2}∕n\right]}$ is used to assess the goodness-of-fit of the power-law scaling, where *d_i_* is the deviation between the observed value and the estimated one.

### ANOVA statistical test

The single-trial analysis results obtained from 215 samples of the math-gifted group and 252 samples of the control group were examined statistically using the one-way analysis of variance (ANOVA) in the Matlab Statistics Toolbox, with group (gifted/control subjects) serving as the between-subjects factor. At the nodal level of the graph-theoretical analysis, clustering coefficient and node betweenness centrality of each cortical vertex were statically tested by the one-way ANOVA. Moreover, edge betweenness centrality was tested as well for 30 × 30 links connecting frontal–parietal nodes. The Bonferroni Corrections were used in the multiple statistical tests, with significance level set to 0.05. At the global level of the functional network, the ANOVA was conducted on modularity and characteristic path length, respectively. Additionally, the relevant fitting parameters of PLIs in the power-law model from the single-trial analytical results were statistically compared between the two groups. For the behavioral data, the AVOVA tests were used to identify the group difference in task performances in terms of accuracy and response time.

## Results

### Behavioral measure of task performance

In the deductive reasoning task, the math-gifted group has outperformed the control group in average response accuracy (mean ± SD: 75.14 ± 12.58% in the math-gifted group and 68.20 ± 15.29% in the control group). Regarding the reaction time of correct response, significant group difference (*p* = 0.0036) has been observed in the task, in which the math-gifted adolescents showed shorter reaction time than the controls (mean ± SD: 831 ± 536 ms in the math-gifted group and 994 ± 655 ms in the control group).

### Enhanced functional integration in the gamma cortical network

**Figure 3:**
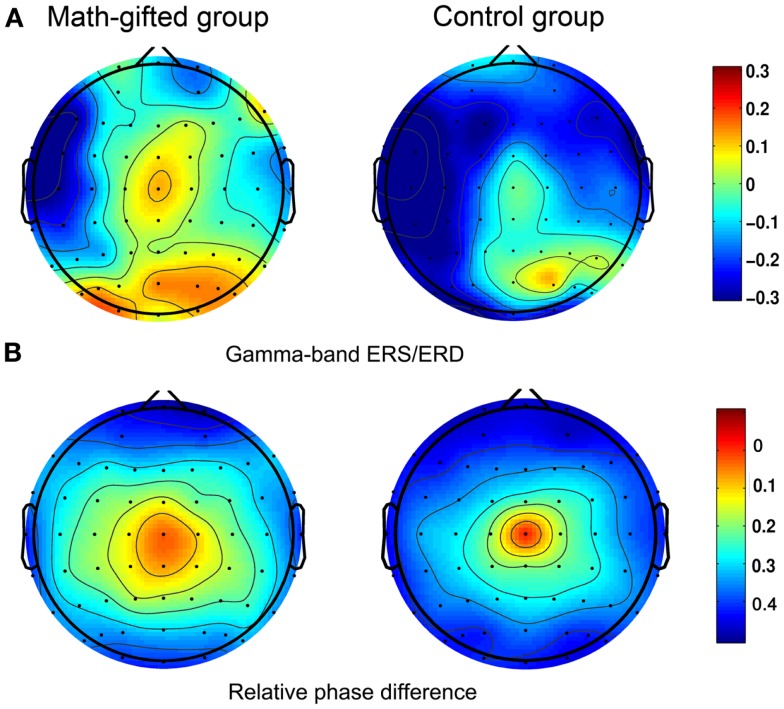
**Scalp activities in spectral power and relative phase difference**: **(A)** task-induced ERS/ERD at gamma frequency band; **(B)** relative phase topologies, in which the electrode at central location is used as the reference. The left column is from the averaged value of the math-gifted subjects, and the right column is from the control subjects.

The ERS/ERD based brain topological maps show that the gamma-band response induced by the deductive reasoning task is mainly distributed in the prefrontal, frontal, sensorimotor, parietal, and occipital regions. The math-gifted group in particular has higher gamma-band ERS in the central sensorimotor regions as compared with the average-ability subjects (Figure 3A). Corresponding to this result, relatively extensive brain regions with small phase difference are discovered in the math-gifted group, as shown in the phase topologies from the averaged values of the subjects in the time window of data analysis (Figure 3B).

**Figure 4:**
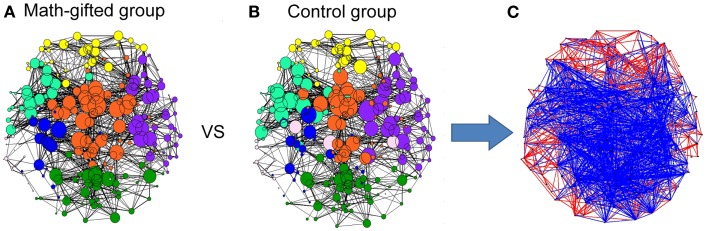
**Gamma neurocognitive network topologies and between-groups difference in synchronized node pairs**: **(A)** network topology derived from the averaged PLV matrix of the math-gifted subjects; **(B)** network topology derived from the averaged PLV matrix of the control subjects. Each node represents a cortical vertex, and the size of node is proportional to the degree of node. The color of node indicates the membership of topological module, which is segmented by the Louvain method for functional community detection; **(C)** the difference of phase synchronization between the PLV matrices of the math-gifted and control groups. As compared to those of the control group, the blue edges represent the increased synchronizations of the math-gifted group, and the red edges are the decreased synchronizations.

**Figure 5:**
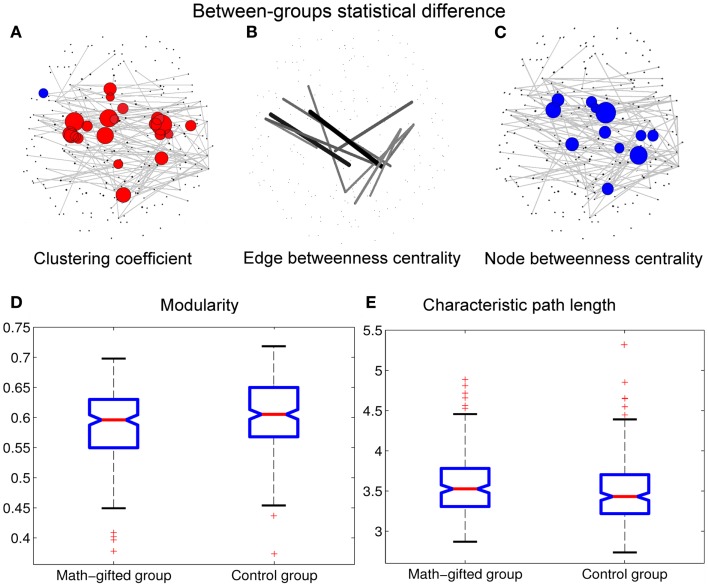
**Between-groups statistical differences of topological parameters: when the topological parameters of the math-gifted subjects are compared with those of the control subjects, the statistical differences are shown in (A) the red nodes with significantly higher clustering coefficient and the blue nodes with significantly lower clustering coefficient (adjusted *p* < 0.05/248), (B) the links with significant higher edge betweenness centrality (adjusted *p* < 0.05/900), (C) the cortical vertices with significantly lower node betweenness centrality (adjusted *p* < 0.05/248), (D) the significantly lower modularity of global network (*p* < 0.01), and (E) the significantly longer characteristic path length (*p* < 0.05)**. The size of node/line corresponds to log *p* value for an ANOVA test with the null hypothesis that between-groups difference is zero.

From the graph-theoretical analysis results of the gamma cortical network, the basic neurocognitive network topologies of the two groups are primarily composed of the prefrontal, frontotemporal, parietal, occipital, and fronto-parietal modules. With the same connection density employed in the two groups, the gamma synchronization network in the math-gifted group shows an expanded fronto-parietal module that integrates more cortical vertices in frontal, parietal, and sensorimotor regions and the relatively shrinking frontotemporal modules, by using the Louvain method for functional community detection (Blondel et al., 2008). In the comparison between the PLV matrices from the two groups, the intensively increased synchronized node pairs in the gamma cortical network of the math-gifted subjects focus on the fronto-parietal cortical regions, accompanied with the node pairs with decreased synchronization in prefrontal, temporal, and occipital regions (Figure 4). Moreover, the ANOVA results for testing the between-groups difference in the individual nodes show that the math-gifted adolescents have significantly high clustering coefficients on the nodes in the fronto-parietal module (adjusted *p* < 0.05/248), especially in the sensorimotor area (Figure 5A), which means enhanced local interconnectivity or cliques among the neighbors of the nodes in fronto-parietal cortical area and correlates with higher local efficiency of information transfer and robustness of fronto-parietal network (Bullmore and Sporns, 2009; Power et al., 2010; Kitzbichler et al., 2011).

In the math-gifted brain, the expanded fronto-parietal functional module and enhanced connectivity of the frontal–parietal network are associated with the emergence of more connections between structurally separated frontal and parietal cortical vertices. The ANOVA results indicate that some frontal–parietal links show significantly higher edge betweenness centrality in the cortical network of the math-gifted subjects (adjusted *p* < 0.05/900), suggesting the enhanced role of “connector bridges” of the frontal–parietal connections (Figure 5B). The increased direct connections in the fronto-parietal network can make the distant nodes be linked through relatively few intermediate steps, which supports the straightforward information communication and promotes the capacity of parallel information transfer of the fronto-parietal network. Specifically, more fronto-parietal “connector bridges” would decrease the dependence of inter-area information communication on the “connector hubs” and increase the robustness of the gamma network even in the case of the hub lesion. As shown in Figure 5C, the cortical vertices with significantly lower node betweenness centrality (adjusted *p* < 0.05/248), i.e., decreased role of “connector hubs,” in the math-gifted brain are found being located at the central sensorimotor area, involving some of the cortical vertices in premotor and primary motor regions (Figure 5C).

Besides, the ANOVA analysis of the global network further demonstrates that the math-gifted adolescents have significantly lower modularity in the global network topology as compared to the average-ability subjects (Figure 5D), which reflects the highly integrated configuration pattern at the level of global topology. However, the longer characteristic path length in the math-gifted group indicates the less economical network configuration, which might be caused by the fixed connection density used in the network analysis that would lead to the disconnected nodes in prefrontal, temporal, and occipital regions (Figure 5E) (Table 1).

**Table 1:** **Between-groups *F*-tests for differences in graph measures of global network topology with fixed connection density: SS, sum of squares; df, degrees of freedom; MS, mean square**.

	Source	SS	df	MS	*F*	*P*
Modularity	Group	0.0131	1	0.0131	11.09	*p* < 0.01
	Error	0.5486	465	0.0012		
	Total	0.56169	466			
Characteristic path length	Group	0.0852	1	0.0852	3.91	*p* < 0.05
	Error	10.1389	465	0.0218		
	Total	10.2241	466			

### Prolonged phase-locking duration and increased lability of network reorganization

**Figure 6:**
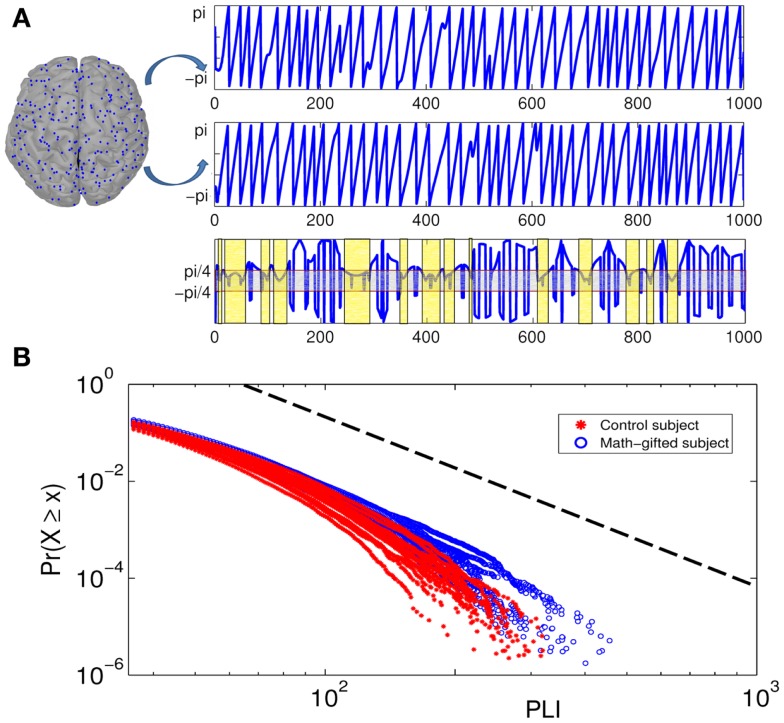
**Illustration of phase-locking duration between pairs of cortical signals and power-law distribution diagrams of PLIs at gamma frequency**: **(A)** the top of the diagram is the phase signals of the two cortical signals from a frontal–parietal node pair. The bottom of the diagram is the time curve of the phase difference between them. The horizontal axis is time course and the vertical axis represents phase difference between the cortical signals. The light gray box contains the region in which the phase signals are synchronized, and the light yellow boxes represent the PLIs within which the synchronization is unintermittent. **(B)** Cumulative distribution function of PLI ( >35 ms) plotted on logarithmic axes. The blue fitting curves are derived from all the math-gifted subjects and the red fitting curves are derived from all the control subjects. The horizontal axis is PLI and the vertical axis is cumulative probability density. The black dotted line represents a power-law rule with exponent *α* = 3.

From the result of PLI analysis, the increased inter-module connections of fronto-parietal network can be attributed to stable phase dynamics of synchronization oscillation between distant brain regions (Thatcher et al., 2009a). Figure 6A illustrates the episode phase-locks between a pair of frontal–parietal cortical signals and the time-varying process of phase-lock/shift (synchronization/desynchronization) between them. Compared with the average-ability subjects, the longer mean phase-locking duration in the math-gifted adolescents represents a wider range of stable patterns of PS in the time domain, which supports straightforward communication and functional coupling of the frontal–parietal cortical areas (Figure 8A).

**Figure 7:**
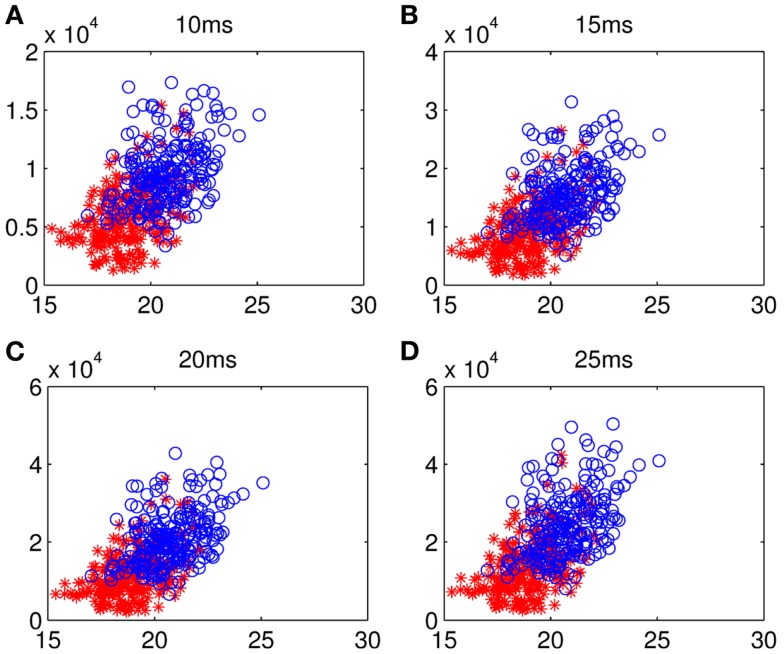
**Scattergrams of frontal–parietal PLI and network lability: the blue circles represent the samples from all the math-gifted subjects and the red asterisks are the samples derived from all the control subjects**. The horizontal axis is mean value of the frontal–sensorimotor, sensorimotor–parietal, and frontal–parietal PLIs and the vertical axis represents fronto-parietal network lability in **(A)** 10-ms, **(B)** 15-ms, **(C)** 20-ms, and **(D)** 25-ms.

Although too long phase-locking duration has been surmised to lead to the lack of flexibility of neural activity (Thatcher et al., 2008), Figure 7 shows a tendency that the prolonged fronto-parietal PLI accompanies with the increase of fronto-parietal network lability. The results of the LDA between the two groups with classification accuracies of 0.8026, 0.7997, 0.7831, and 0.7811, corresponding to different time intervals, indicate that the math-gifted brain could be characterized by longer PLI and higher lability in the fronto-parietal network reorganization, especially for the relatively rapid change in the 10 and 15 ms intervals (Figures 7A,B). From the samples of the math-gifted subjects, the long mean PLI helps information processing of network and the extensive adjustment of fronto-parietal connections indicates the widespread connection reorganization to adapt to temporal binding for cognitive event. The phase-lock mechanism in the math-gifted brain represents an optimized synchronization pattern in functional binding of fronto-parietal network, because it simultaneously supports the phase “stability” of functional coupling and the “flexibility” of network connection reorganization.

### Power-law distribution of large phase-locking durations

The coordination relationship in functional binding of fronto-parietal network can be explained by the power-law distribution of PLIs. Based on a plenty of PLI samples from the trial concatenation for each subject (the sample size *n* > 10^6^) (Table 2), Figure 6B depicts the cumulative distribution functions *P*(*x*) of the PLIs of all the subjects when *x* is >35 ms. It can be seen that each PLI distribution follows the power-law rule (the standard deviation of the estimated values *R*_e_ < 0.5%), which is manifested as an exponential fall-off. It is notable that the obvious difference between the two groups is presented in the distribution tail that represents large but rare synchronization and critical behavior as well (Clauset et al., 2009; Kitzbichler et al., 2009).

**Table 2:** **Basic parameters of the power-law fitting of individual PLI samples between 30 × 30 node pairs from trial concatenation: *n*, sample size; ⟨x⟩, mean value of samples; *x*_max_, maximum PLI; ${\hat{x}}_{\min}$, estimated minimum PLI of power-law distribution interval; $\hat{\alpha }$, estimated power-law exponent; *n*_tail_ = [${\hat{x}}_{\min}$, *x*_max_]; *R*_e_, standard deviation of estimated values**.

	*n*	⟨x⟩	*x*_max_	${\hat{x}}_{\min}$	$\hat{\alpha }$	*n*_tail_	*R*_e_ (×10^−2^)
**MATH-GIFTED SUBJECT**							
01	311242	20	316	36	2.89	280	0.31
02	316268	21	438	37	2.83	401	0.26
03	325099	22	332	37	2.86	295	0.27
04	205294	23	376	33	2.87	343	0.33
05	335295	21	355	37	2.80	318	0.23
06	201462	20	347	35	2.82	312	0.25
07	268759	19	334	33	2.92	301	0.29
08	260084	20	265	36	2.9	229	0.29
09	334978	21	432	35	2.92	397	0.30
10	390596	21	456	37	2.86	419	0.21
11	568511	21	401	36	2.85	365	0.28
Mean value	319781	21	368	37	2.86	333	0.27
**CONTROL SUBJECTS**							
01	318080	18	258	31	2.90	227	0.32
02	322137	19	296	35	2.94	261	0.30
03	252469	18	290	31	2.95	259	0.32
04	313898	17	261	31	2.90	230	0.36
05	307197	20	292	36	2.85	256	0.28
06	321789	19	355	34	2.92	321	0.27
07	312976	21	319	36	2.81	283	0.25
08	383190	19	289	34	2.92	255	0.32
09	297221	17	221	29	2.93	192	0.41
10	401429	20	316	34	2.88	282	0.33
11	396750	19	281	33	2.93	248	0.32
12	362526	19	249	34	2.94	215	0.26
13	275928	19	331	33	2.91	298	0.31
Mean value	328122	19	289	33	2.9	255	0.31

**Figure 8:**
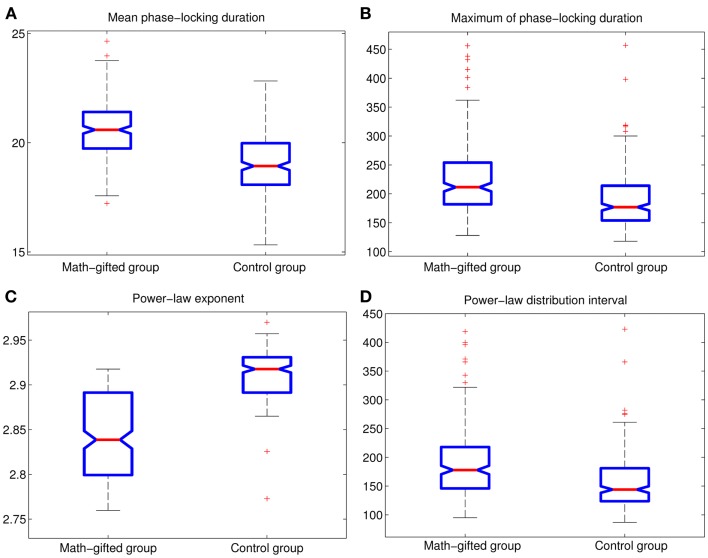
**Between-groups AVOVA tests for basic power-law fitting parameters of PLIs from signal-trial data (*p* < 0.01): (A) mean PLI; (B) maximum of PLI; (C) power-law exponent; (D) power-law distribution interval of PLI**.

The basic parameters of the power-law fitting from the single-trial data provide statistic evidence for the difference between the two groups. Corresponding to the higher maximum PLI values, the math-gifted subjects show wider power-law interval of PLIs distribution, i.e., the critical interval, and lower power-law exponent (Figures 8A–D) (Table 3). In the expanded critical interval, large synchronization durations ( >35 ± 3.2 ms) play an important role in maintaining the inter-module connectivity temporally, although they form a small proportion in the total PLI samples. At the same time, the synchronizations in the critical interval are surmised to be tuned to the critical point of state transition, which could make the fronto-parietal synchronizing state “metastable.” Additionally, the lower power-law exponent of the math-gifted brain could be viewed as an indicator of higher intrahemispheric frontal–parietal connectivity, as it is found to be correlated to stronger structural connectivity (Kitzbichler et al., 2009).

**Table 3:** **Between-groups *F*-tests for differences in power-law fitting parameters of PLIs: SS, sum of squares; df, degrees of freedom; MS, mean square**.

	Source	SS	df	MS	*F*	*P*
Mean phase-locking duration	Group	261.8558	1	261.8558	142.1430	*p* < 0.01
	Error	856.6231	465	1.8422		
	Total	1.1185e + 003	466			
Maximum of phase-locking duration	Group	1.3654e + 005	1	1.3654e + 005	41.4677	*p* < 0.01
	Error	1.5311e + 006	465	3.2927e + 003		
	Total	1.6677e + 006	466			
Power-law exponent	Group	0.1901	1	0.1901	131.0518	*p* < 0.01
	Error	0.6746	465	0.0015		
	Total	0.8647	466			
Power-law distribution interval	Group	1.1235e + 005	1	1.1235e + 005	35.5266	*p* < 0.01
	Error	1.4705e + 006	465	3.1624e + 003		
	Total	1.5829e + 006	466			

Critical synchronization can be compatible with the rapid network reorganization in response to temporary perturbation and stimulus, which promotes the adaptive ability of a functional network in spatial reconfiguration of connections (Bassett et al., 2006; Kitzbichler et al., 2009). The adaptive change imposed on a network is realized through local rewiring rules motivated by the activity-dependent synaptic development (Bornholdt and Röhl, 2003). The rich distant connections in fronto-parietal network of the math-gifted brain provide more available links and selection advantage to operate the local rewiring rule, since the adjustment of these connections has been found to be the most salient gamma network change during the adaptive network reconfiguration (Bassett et al., 2006; Kitzbichler et al., 2011). In the math-gifted brain, the phase-locking durations abiding by wider power-law distribution might account for the optimized synchronization pattern of functional binding through achieving a better balance between prolonged PLI and increased network lability.

## Discussion

The paradigms used in the previous studies on math-gifted adolescents or children mostly involved visuospatial imagery tasks that were related to mathematical thinking ability, such as RAPM test and mental rotation. As an essential mathematical skill, a cognitive task of the analytic type (verbal–logical way) was designed in this study for determining whether the previous research results were specific to the mathematical thinking or just the general attributions of problem solving. The logical syllogism used in this experiment is viewed as a basic form of mathematically logical thinking and fills the void of the experimental paradigm in neuroscience studies of mathematical giftedness.

To the best of our knowledge, this is the first time that the individual difference between math-gifted and average-level abilities is investigated through EEG dynamic network analysis. With the highest criticality in the fractal networks of the human brain, the cortical network at the classic gamma frequency is assessed by transforming the scalp-recorded EEG signals into the cortical dipoles. According to the results obtained from the graph-theoretical analysis, the math-gifted adolescents demonstrate a highly integrated fronto-parietal network that is supported by the prolonged gamma binding-by-synchrony activity among discrete neuronal assembles, which is in line with the results of the previous fMRI studies and the P-FIT model of reasoning. Furthermore, as the prolonged periods of phase-locking are more likely to occur between the processes within the same functional module (Kitzbichler et al., 2009), the fronto-parietal PLIs in the math-gifted brain might be the consequence of strong structural connectivity of fronto-parietal network. On the other hand, the math-gifted subjects recruited in our experiment might have more practice with this kind of reasoning task by virtue of their exposure to more education. The mental training-related effect might lead to the changes of neuroelectric activities in phase-locking. That is, perhaps the performances of the math-gifted adolescents in gamma synchronization are not solely due to greater innate ability.

Functional connectivity of the phase coherent network is positively related to the phase-locking duration and stability of phase dynamics. In the context of temporally stable fronto-parietal connectivity in the math-gifted brain, the theory of critical dynamics is applied to the realistic data from the high-order cognitive task through the analysis of single-trial samples, which constructs an association between the enhanced functional connectivity and the highly adaptive reconfiguration of the fronto-parietal network in the math-gifted brain. From the perspective of criticality, the existence of power-law distribution of PLIs in the brain puts the large synchronization on a “metastable island”; that is, the longer the PLI is, the higher the desynchronization possibility will be (Werner, 2007). The large-sample EEG study conducted in 378 children and adolescents (Thatcher et al., 2008) has suggested that, the “optimal” balance between phase-locking duration and phase-shifting duration benefits the effective allocation of neuronal resources, and is related to high intelligence level that has been consistently considered as a basic factor of mathematical giftedness. The cortical network study in this paper supports the opinion that the math-gifted adolescents can use the well-allocated phase-lock resources to facilitate the functional binding in the fronto-parietal cortices, since the temporal binding between neuronal assembles depends on the transient coupling and adapts to the timely connection redistribution of network. Empirical studies have demonstrated that the significant gamma network reorganization is affected by the motor task, working memory task, cognitive effort, etc. (Bassett et al., 2006; Kitzbichler et al., 2011). In the math-gifted brain, the optimized phase-lock pattern in functional binding would make the synchronizing network flexibly compatible to varying cognitive requirement of the reasoning process. Except the neural correlates of mathematical giftedness, there is evidence that phase-locking and phase-shift durations in EEG low-frequency intervals are significantly different in people with Autism Spectrum Disorder (ASD), with longer periods of phase-lock and fewer phase-shifts (Thatcher et al., 2009b). In addition, the individuals with ASD also have been found showing the abnormal functional connectivity between some regions in default model network (Assaf et al., 2010). As there are frequent reports of the relevance between people with ASD and high mathematical ability, the phase-locking mechanisms in the both populations might follow the similar distribution rule. Perhaps in another aspect of phase-locking duration and network reconfiguration, too long period would also lead to the decreased flexibility of adaptive network reconfiguration, because of the reduced resources available to be operated by the phase-shift mechanism (Thatcher et al., 2008). Due to the difference in network wiring, the locally over-connected functional network in the brain might be related to the deficits seen in ASD.

The optimized synchronization pattern of the fronto-parietal network also plays a key role in information processing. The prolonged fronto-parietal phase-locking durations distributed in a wider critical interval indicate that some optimizations of information processing would occur simultaneously. Firstly, the generally prolonged phase-locking durations enhance the global synchronization of the gamma network through a widespread stability of phase dynamics, which could increase the capacity of information storage of the network. Secondly, the phase-locking duration at a critical state supports effective information communication between neuronal assembles because the long synchronization leads to efficient information transmission. Finally, when the synchronizing activity is maintained at a critical state, it would decrease the stability of the connection but increase the adaptiveness of the network for timely reorganization of connections. In conclusion, the optimizations of the fronto-parietal synchronization enhance the information processing of the math-gifted brain during the deductive reasoning task, and further support the exceptional logical thinking ability of math-gifted adolescents.

## Author Contributions

John Q. Gan and Haixian Wang designed the research and provided analytic tools; Li Zhang conducted experiment, analyzed data, and wrote the paper; John Q. Gan and Haixian Wang improved the paper.

## Conflict of Interest Statement

The authors declare that the research was conducted in the absence of any commercial or financial relationships that could be construed as a potential conflict of interest.
